# Experimental Analysis of the Low-Velocity Impact and CAI Properties of 3D Four-Directional Braided Composites after Hygrothermal Aging

**DOI:** 10.3390/ma17133151

**Published:** 2024-06-27

**Authors:** Yuxuan Zhang, Hanhua Li, Shi Yan, Xin Wang, Yue Guan, Changmei Du, Lili Jiang, Junjun Zhai

**Affiliations:** 1 Department of Engineering Mechanics, Harbin University of Science and Technology, Harbin 150000, China; 15682852313@163.com (Y.Z.); wangxiniiiiii@outlook.com (X.W.); 15155944384@163.com (Y.G.); 18306393902@163.com (C.D.); 2 Department of Engineering Mechanics, Beijing Institute of Astronautical Systems Engineering, Beijing 100076, China; 13b918023@hit.edu.cn; 3 College of Civil Engineering and Architecture, Xiamen City University, Xiamen 361008, China; jianglili@xmcu.edu.cn; 4 College of Aeronautics and Astronautics, North China Institute of Aerospace Engineering, Langfang 065000, China; junzhai726@nciae.edu.cn

**Keywords:** hygrothermal aging, 3D four-directional braided composites, low-velocity impact (LVI), compression after impact (CAI), C-scan, SEM, DIC, TGA

## Abstract

Three-dimensional braided composites (3D-BCs) have better specific strength and stiffness than two-dimensional planar composites (2D-PCs), so they are widely used in modern industrial fields. In this paper, two kinds of 3D four-directional braided composites (3D4d-BCs) with different braided angles (15°, denoted as H15, and 30°, denoted as H30) were subjected to hydrothermal aging treatments, low-velocity impact (LVI) tests, and compression after impact (CAI) tests under different conditions. This study systematically studied the hygroscopic behavior and the effect of hygrothermal aging on the mechanical properties of 3D4d-BC. The results show that higher temperatures and smaller weaving angles can significantly improve the moisture absorption equilibrium content. When the moisture absorption content is balanced, the energy absorption effect of 3D4d-BC is better, but the integrity and residual compression rate will be reduced. Due to the intervention of oxygen molecules, the interface properties between the matrix and the composite material will be reduced, so the compressive strength will be further reduced. In the LVI test, the peak impact load of H15 is low. In CAI tests, the failure of H15 mainly occurs on the side, and the failure form is buckling failure. The main failure direction of H30 is 45° shear failure.

## 1. Introduction

With the continuous progress of human industry, as the representative of applied materials, the development of composite materials has been widely focused on and applied by people. Three-dimensional braided composites (3DBCs), as an alternative to traditional two-dimensional laminates composites (2DLCs), have been widely used in the modern aerospace industry, automobile manufacturing, shipbuilding, medical equipment [1,2,3], and other fields due to their better integrity, higher specific strength, better thermal and chemical stability, and excellent fatigue resistance [4,5,6,7,8]. However, when 3D-BC bursts in the outdoor environment due to the influence of environmental factors such as high temperatures, hygrothermal conditions, salt spray, and ultraviolet rays [9,10,11,12], it will inevitably have an important impact on its mechanical properties, resulting in reversible or irreversible physical or chemical changes [13,14].

At present, a considerable number of research reports have confirmed that the hygrothermal environment will affect the physical and mechanical properties of composite materials, and the main reason for the performance decline is the hygroscopic behavior of the matrix [15,16,17,18]. Specifically, hygroscopic behavior will cause effects such as swelling, plasticization, creep, increased stress relaxation, lower glass transition temperatures (Tg), lower mechanical strength, and lower elastic moduli [13,19,20,21,22,23,24,25]. For example, Baoming Wang et al. [26] studied the effects of hygrothermal and salt spray environments on the properties of composite materials. It was found that water absorption during aging resulted in tiny cracks at the fiber–resin matrix interface. These microcracks are the main reason for the decrease in flexural strength and impact strength of composites. Alessandro et al. [27] found that the tensile strength and fracture toughness of polyethylene terephthalate matrix composites were significantly reduced under hot and humid conditions. Karbhari et al. [28] conducted relevant studies on the hygroscopic dynamic properties of resin-based composites in a seawater environment. The study found that the diffusivity of the liquid in the composite increased with the increase in aging ambient temperatures and showed changes based on the post-curing level and the change in the network with time. Zhou [29] and Wang et al. [30] further classified the water in the resin into type I or Type II according to the difference in the bond and/or properties. Type I has a low thermal activation energy and will exhibit desorption upon reheating. The two different types of water also have different desorption mechanisms. Chin et al. [31] report that water absorption in epoxy resins is essentially Fick’s, with diffusion coefficients in salt solutions almost double those in distilled water. Zhang et al. [32] studied the effects of seawater content and temperature on the properties of carbon fiber-reinforced polymers (CFRPs) using molecular dynamics methods. At the same time, the degradation mechanism of CFRP in a seawater environment was studied comprehensively. Wang and Ghabezi et al. [33,34,35] also conducted relevant research on the long-term durability of CFRP under corrosive environments (artificial salt water, seawater, sea sand concrete environment, and sea-based environment), and they obtained the degradation law and mechanism of long-term performance.

The properties of composite materials mainly depend on the matrix, reinforcement (fiber, etc.), and the bond between them. Although carbon fiber is inert to water, the resin matrix is affected by water absorption and the reaction of the polymer network. Therefore, the study on the hygroscopic behavior and hygroscopic mechanism of composite materials in a hygrothermal environment is still the part that researchers are concerned about. Apicella et al. [36,37] proposed three main adsorption modes: (a) the dissolution of water molecules in the polymer; (b) water molecules are absorbed onto surfaces in the free volume of the glassy network; and (c) the bonding between hydrophilic groups on the polymer chain and water molecules. The hydrolysis of polymer networks and fiber–matrix interfaces depends on not only the exposure time but also the type of aqueous solution [38,39,40,41]. Although there have been many studies on different matrix types, the water absorption model based on Fick’s law [42,43,44] and the Langmuir model [45] is generally considered to be the best method for studying the water absorption mode of resin matrix composites.

In addition to experimental studies, researchers have also paid attention to numerical simulations [46,47]. It has become the main work of researchers to establish a multi-scale numerical model to simulate the hygroscopic behavior of composite materials and related experiments. For example, Zhou et al. [48] developed a numerical model based on the Hashin failure criteria to describe the mechanical behavior of CFRP laminates. Mohammad Rezasefat et al. [49] established a material model based on continuous damage mechanics. The model uses the strength of the Puck surface as the damage initiation criterion, and it proves the validity of the damage model at the unit level. Gao et al. [5] adopted the Linde and Hashin failure criteria to establish a numerical model to analyze the bending properties and failure mechanism of composite materials.

Based on this, although many scholars have studied the damage mechanism of composite materials under different service environments, the study on the influence of mechanical properties of 3D four-directional braided composites (3D4d-BCs) under complex environments is still insufficient. Based on this, the study designed three different hygrothermal aging conditions (40 °C-soak, 70 °C-soak, and 70 °C/85%RH) and aging times (500 h, 1000 h, and 2000 h) to simulate a real hygrothermal environment. A variety of experimental analysis methods were used to analyze the aging behavior and its influence on mechanical properties. Specifically, thermogravimetric analysis (TGA) was used to analyze hygroscopicity; a scanning acoustic microscope (C-scan) was used to observe and calculate internal damage after the low-velocity impact (LVI) test and compression after impact (CAI) test; the compression fracture was characterized via scanning electron microscopy (SEM); the compression process was monitored via digital image correlation (DIC). This not only has a certain guiding significance for engineering tests but also has a certain positive significance for further proposing and verifying more efficient and realistic numerical models. The main research aspects of this experiment are as follows:

(1) The hygrothermal aging of 3D4d-BC was performed to analyze the hygroscopic behavior and thermal stability of 3D4d-BC under different hygrothermal aging conditions and different braiding angles (15°/30°);
(2) An LVI test was carried out to analyze the influence of the hygrothermal aging process on the impact resistance of specimens;
(3) A CAI experiment and DIC damage analysis were used to analyze the influence of the hygrothermal aging process on the residual compression properties of the specimens.

## 2. Materials and Process

### 2.1. Materials and Equipment

In this experiment, the 3D4d-BC matrix was made of a thermosetting resin matrix, specifically a TDE-86- epoxy resin matrix. TDE-86 is an alicyclic glycidyl ester type three functional epoxy resin. Its glass conversion temperature (Tg) is about 219.5 °C, and it was cured using a single-component curing agent. Toray T700-SC-1000-50B carbon fiber is used for the prefabricated yarn of 3D4d-BC. The experimental materials were produced by Hubei Feilihua Quartz Glass Co., Ltd. (Jingzhou, China).

Due to errors in the manufacturing process, the volume fraction of all specimens is different. According to statistics, the fiber volume content of specimens with a braided angle of 15° ranges from 60.91% to 62.85%, with an average of 62.02%. For specimens with an angle of 30°, the fiber volume content ranges from 63.47% to 64.97%, with an average of 63.98%. The fineness of the single-strand carbon fiber is 800 tex.

**Figure 1:**
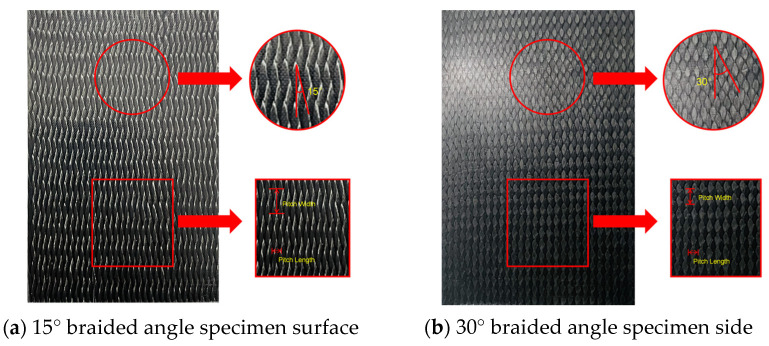
Three-dimensional four-directional braided composite specimens.

In this paper, the matrix is denoted as “H”, H15 represents 3D4d-BC with a braided angle of 15°, and H30 represents 3D4d-BC with a braided angle of 30°. The length of the pitch varies according to the braided angle. The specimen’s size is 120 mm × 80 mm × 5 mm with an error of 0.5 mm. The relevant parameters of materials are shown in Table 1, and the photos of specimens are shown in Figure 1.

### 2.2. Hygrothermal Aging

During the implementation of the hygrothermal test, the main test equipment is a constant temperature water bath and a constant temperature and humidity testing machine. The liquid used in the hygrothermal aging process is distilled water. The equipment’s name and model parameters are shown in Table 2.

The hygrothermal aging of 3D4d-BC was carried out, and the test process was as follows: H15 and H30 were tested for 500 h, 1000 h, and 2000 h under constant humidity conditions of 40 °C-soak, 70 °C-soak, and 70 °C/85% RH (relative humidity). Among them, two groups of parallel tests were conducted in each group, and two groups of specimens in a room temperature environment (this means unaged) were set as the control group. The hygroscopic content Mt is calculated using statistical sample mass changes, and the calculation formula is shown in Formula (1):

(1) $$M_{t}=\frac{M_{i}-M_{i-1}}{M_{0}}\times 100\%$$

where “*M_t_*” in Formula (1) means hygroscopic content at time “*t*”; “*M_i_*” is the mass of the test piece measured on day “*i*”, *i* = 1, 2, 3, …; and “*M*_0_” is the mass before hygrothermal aging.

### 2.3. Low-Velocity Impact Test (LVI)

The main experimental instruments used in the impact experiment are a drop hammer impact testing machine, custom fixture, spherical punch, and scanning acoustic microscope (C-scan). The C-scan equipment type is Sonoscan-D9500 (Santa Clara, CA, USA). The LVI test was carried out on the Instron-9250 HV (Norwood, MA, USA). In LVI, the standard impact energy selected of 30 J, the mass of the single-head ball punch was 0.145 kg, and the total mass of the impact platform was 7.29 kg.

The equipment names and model parameters of the drop hammer impact testing machine and the C-scan instrument are shown in Table 3.

The impact specimens are H15 and H30 after the hygrothermal aging process. The test specimen numbering rules are as follows: “H-braided angle—hygrothermal aging time—hygrothermal aging environment”; “R” means room temperature environment; “RH” means relative humidity. For example, “H15-500-70 °C/85% RH” is a 3DBC specimen with a braided angle of 15° that has been subjected to hygrothermal aging for 500 h at 70 °C/85% RH. The numbers of all specimens are shown in Table 4. It should be pointed out that each group of impact test pieces is used twice in parallel tests, and the average value is calculated as the calculation result for statistics.

After the LVI test, the C-scan was used to detect the internal damage area of the specimen. Since the emission direction of the ultrasonic wave in the C-scan is perpendicular to the scanning plane, the image obtained after scanning is the superimposed area of the specimen to be scanned. Therefore, scanning can simply and directly obtain the regional shape of the defect damage site.

### 2.4. Compression after Impact Test (CAI)

CAI tests were carried out on the specimens with impact damage on the universal test machine. The universal testing machine is divided into two pressure plates, the lower part is fixed, the upper part of the pressure plate moves down to apply the load, and the maximum applied load is 100 KN. The relative error of the test machine is 1%, and the accuracy of displacement measurements is 0.01 mm. 

During the experiment, the test piece needs to be mounted on the custom fixture so that the left and right sides and the bottom are fixed, and the knitting direction of the test piece is kept upright. The test piece was loaded by displacement at a loading speed of 0.5 mm/min until the specimen collapsed.

## 3. Analysis of Hygrothermal Aging

### 3.1. Mass Change Rate of Different Braided Angles after Hygrothermal Aging Process

**Figure 2:**
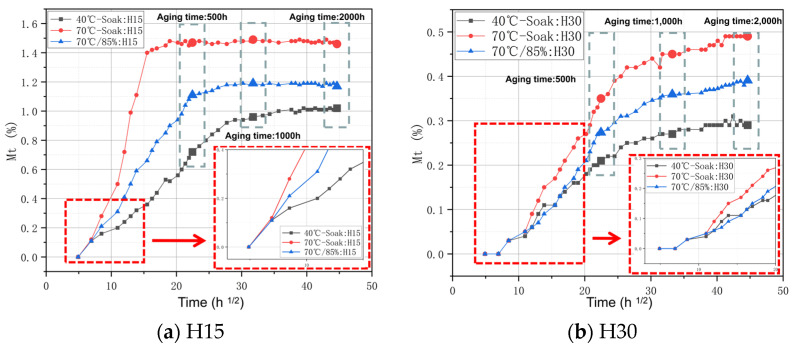
Moisture absorption rate of the same matrix and weave angle under different humid and thermal aging conditions.

Compared with humidity, temperature is the factor that directly affects the hygroscopic behavior of the epoxy resin base. The increase in the weaving angle has little effect on the hygroscopic rate and will directly affect the hygroscopic equilibrium content. The quality change curves of different weaving angles under different conditions are shown in Figure 2. 

On the whole, the hygroscopic content shown in Figure 2 presents an approximately linear relationship with the square root of time, and the overall development follows Fick’s second law:

(1) The moisture absorption rate and moisture absorption equilibrium content of the material with the same braiding angle increase with an increase in temperature. Specifically, H15 is soaked at 70 °C and reaches moisture absorption equilibrium (about 1.5%) at an aging time of 17 d (about 400 h). At 40 °C-soak, the moisture absorption equilibrium (about 1.0%) is reached at an aging time of 40 days (about 960 h). With the increase in temperature, the time to reach the moisture absorption equilibrium is shortened by 58.33%, and the moisture absorption equilibrium is increased by nearly 50%. This is due to the increase in temperature, which directly affects the speed of movement of the water molecules. When the aging temperature is 70 °C, the interaction between the fiber and the matrix will be more affected, resulting in the separation of the two, and more water molecules will enter the gap between the two.
(2) The increase in humidity increases the hygroscopic equilibrium content and hygroscopic rate of H15 and H30. With the increase in humidity, the time to reach the moisture absorption equilibrium content was shortened by 42.86%, and the moisture absorption equilibrium content was increased by nearly 25%. Specifically, H15 when soaked at 70 °C reaches the moisture absorption equilibrium content (about 1.5%) at 17 d of aging time (about 400 h). At 70 °C/85% RH, the moisture absorption equilibrium content (about 1.2%) is reached at 30 d (about 700 h) of aging time;
(3) The larger weaving angle shows a lower moisture absorption equilibrium content throughout the aging process, and it has little effect on the moisture absorption rate. This is because when the weaving angle is larger, the transverse arrangement of the fibers becomes tighter. The hygrothermal aging behavior of resin matrix composites is mainly affected by the epoxy resin matrix and fiber/matrix interface. Therefore, there is a significant difference in the moisture absorption equilibrium content, but it has little effect on the moisture absorption rate.

### 3.2. SEM Analysis

**Figure 3:**
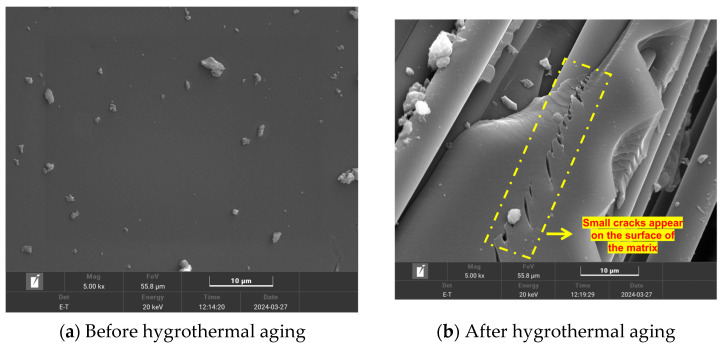
SEM and microstructure images of 3D4d-BC before and after hygrothermal aging.

Since carbon fiber itself does not have hygroscopicity, SEM observations were made on the resin before and after hygrothermal aging, as shown in Figure 3. As shown in Figure 3, the surface of the matrix of the unaged specimen was relatively smooth (Figure 3a). After aging, small cracks and nodules appeared on the surface of the matrix (Figure 3b). This is because, after aging treatment, the fiber/matrix interface is prone to chemical degradation in the presence of water, which reduces the adhesion of the fiber/matrix and enhances the capillary property of the interface. The hygroscopic process creates expansion stress, while the temperature change introduces thermal stress. Under the combined action of expansion stress and thermal stress, these pores gradually expand. With the increase in aging time, microcracks eventually form on the surface of the composite. This is the mechanism of matrix decomposition and degradation. Similarly, our previous studies on the TDE-86 resin base also showed that the matrix would undergo oxidation and hydrolysis to varying degrees during aging treatment [50].

### 3.3. Analysis of Different Braided Angles after Hygrothermal Aging via TGA

**Figure 4:**
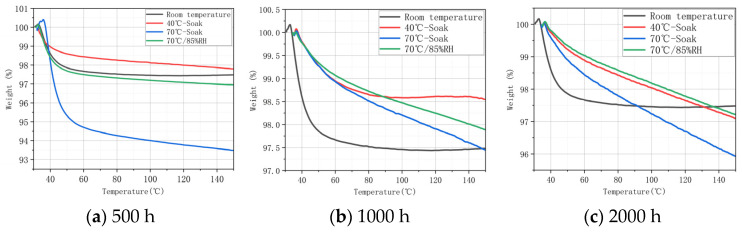
TG curve of H15 after hygrothermal aging.

**Figure 5:**
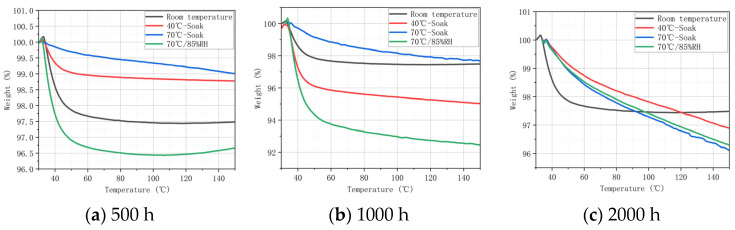
TG curve of H30 after hygrothermal aging.

TGA was applied to the specimen to further analyze the influence of aging conditions on the thermal stability of the matrix. Figure 4 and Figure 5 show the TG curves (TGCs) of H15 and H30 after the aging process in different aging times (0 h, 500 h, 1000 h, and 2000 h) and different aging conditions (40 °C-soak, 70 °C-soak, and 70 °C/85% RH). The initial temperature of TGA was set at 30 °C, and the maximum temperature of epoxy resin heating was 150 °C. In the initial stage of TGA, the weight percentage of the curve slightly exceeded 100% because the heating density of the gas around the crucible decreased after heating, and the weight gain caused by the decrease in buoyancy was negligible.

As shown in Figure 4, after 500 h of aging treatment, the weight decreased significantly at roughly 40 °C. The curve for 70 °C-soak is significantly smaller than in other conditions. This may be because the specimen itself did not completely dried, and the error was caused by a large proportion of water evaporation after the temperature increased. TGC mainly observes the trend of its weight change, and this error will not affect the qualitative analysis. H15 under 40 °C-soak conditions reaches equilibrium when heated to 80 °C (Figure 4b). H15 in the three aging conditions (40 °C-soak, 70 °C-soak, and 70 °C/85%RH), even when heated to 150 °C, still does not reach equilibrium, and its quality is still declining (Figure 4c). This shows that under the same aging conditions, the longer the time, the longer the time it takes for the epoxy resin matrix to completely lose water.

As can be seen from Figure 5, the increase of braiding Angle will have an effect on thermal water loss. Since the carbon fiber itself does not participate in the water absorption process, the matrix and braiding process will also have a certain impact on the thermal stability. The mass decline rate of H30 is 70 °C-85% RH > 40 °C-soak > 70 °C-soak.

## 4. LVI Test and Experimental Results

### 4.1. Analysis of Impact Behavior Response

The LVI performance is analyzed by a “Time–Load” Curve and “Displacement–Load” Curve. By comparing the curves of different hygrothermal aging conditions under the same treatment time, the influence of different hygrothermal aging on the specimen strength was further analyzed. The peak loads of H15 (#1) and H30 (#11) without aging treatment are close to each other in the LVI test. It shows that the change in weaving angle has little effect on the impact performance.

**Figure 6:**
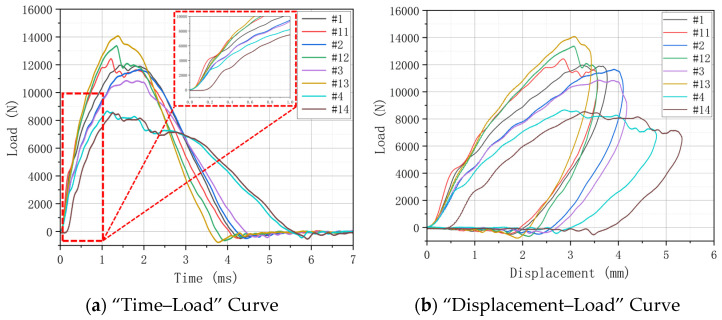
“Time–Load” Curve and “Displacement–Load” Curve under different hygrothermal aging conditions for 500 h.

**Figure 7:**
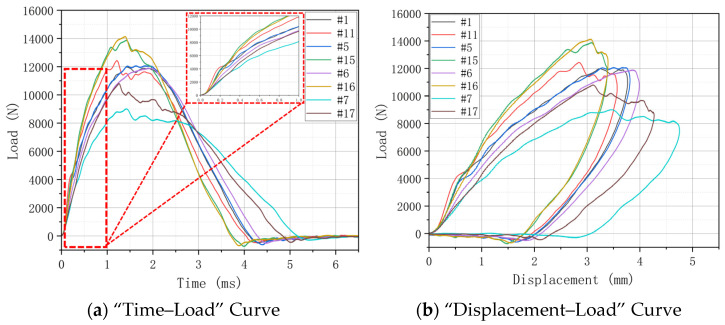
“Time–Load” and “Displacement–Load” Curve under different hygrothermal aging conditions for 1000 h.

**Figure 8:**
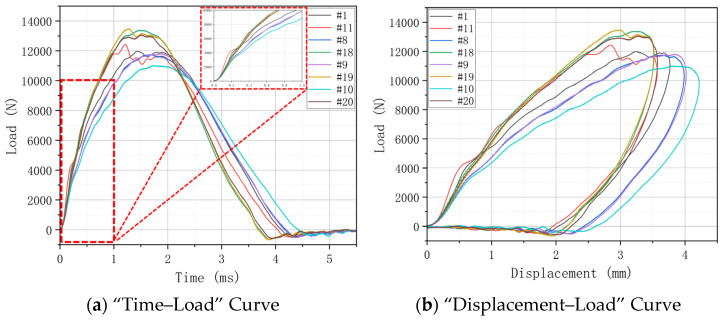
“Time–Load” and “Displacement–Load” Curve under different hygrothermal aging conditions for 2000 h.

On the whole, the peak load increases slightly after aging treatment. This shows that after aging treatment, the ductility of the specimen is increased with the increase in water molecules entering the interior of the composite. At the same time, the epoxy resin matrix after water absorption also provides a certain energy absorption effect for the material when it is impacted, and it increases the overall impact response of the specimen. However, because the integrity of the specimen after aging treatment will become worse, although it has higher impact resistance, its residual compression performance will be reduced, and the overall strength will be reduced. Figure 6, Figure 7 and Figure 8 show the “Load–Time” Curve and “Displacement–Load” Curve under different aging process times.

As shown in Figure 6a, in the process of hygrothermal aging, the temperature has little influence on the impact strength of the specimen, while humidity has a greater influence on the specimen. In the LVI test, the peak load of H15 is smaller than that of H30. This is because, after the aging treatment, H15 absorbs more water, which further leads to the weakening of its structural bearing capacity. The peak load of H30 is about 14,000 N at 70 °C-soak, and about 9000 N at 70 °C/85% RH soak, with a decrease of about 35.71%. The peak load of H15 dropped from about 11,000 N to about 9000 N, a decrease of about 18.18%. The peak load of H30 at 40 °C-soak is about 13,000 N, and its decrease is only 7.14%.

Figure 6b: For H15 and H30, the sequence of factors that cause greater internal damage is 70 °C/85% RH > 70 °C-soak > 40 °C-soak. Therefore, humidity has a certain effect on the hygroscopic properties of the material, and it has a greater effect on the strength properties of the material. In particular, it can reduce the overall strength and affect the adhesion between layers.

The trends and conclusions shown in Figure 7 are basically consistent with the above analysis. After 1000 h of aging time and using 70 °C-soak, the peak load of H30 is about 14,000 N and about 11,000 N at 70 °C/85% RH, with a decrease of about 21.43%. The peak load of H15 drops from around 12,000 N to around 9000 N, a drop of about 25%. In the three aging conditions, the damaged area of the LVI test ranges from large to small: 70 °C/85% RH > 70 °C-soak > 40 °C-soak.

There is a small reduction in the peak load in Figure 8. This is because, after the 2000 h aging treatment, the epoxy resin matrix is affected by water molecules, resulting in a further reduction in the strength of the material. However, on the whole, the LVI test shows the same shock response law as shown above.

### 4.2. Damage of Impact Analysis by C-Scan

After the LVI test, the proportion of the damaged area inside the specimen was calculated, and the results are shown in Table 5: #1–#10 is H15, and #11–#20 is H30. As shown in Table 5, under the same aging condition, the proportion of the internal damage area measured at 0.15 mm after impact is H30 > H15 from large to small. Therefore, under the same aging condition, a greater braided angle could result in a greater internal damage area.

**Figure 9:**
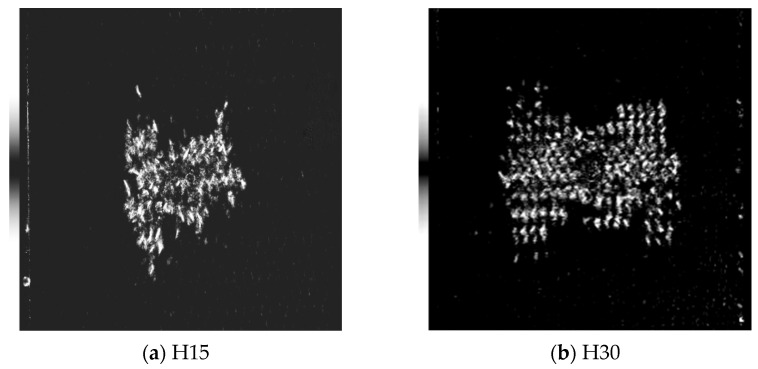
Internal damage of the specimen after the LVI test under room temperature. The scanning area is uniformly 80 mm × 80 mm.

**Figure 10:**
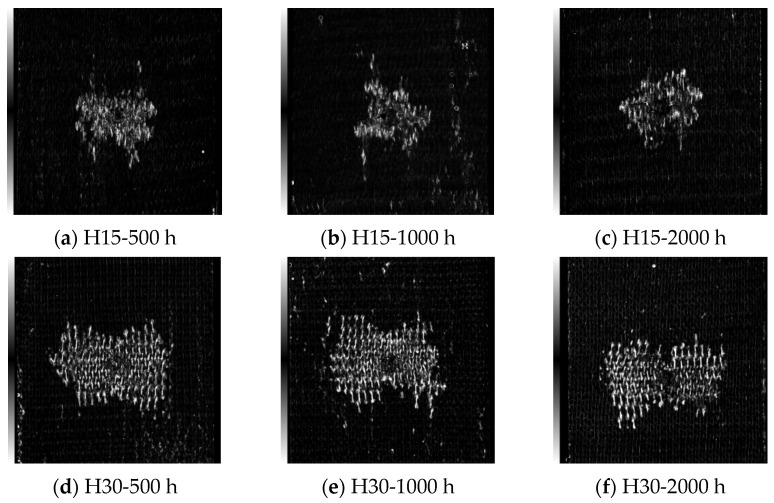
Internal damage of the specimen after the LVI test under 40 °C-soak. The scanning area is uniformly 80 mm × 80 mm.

**Figure 11:**
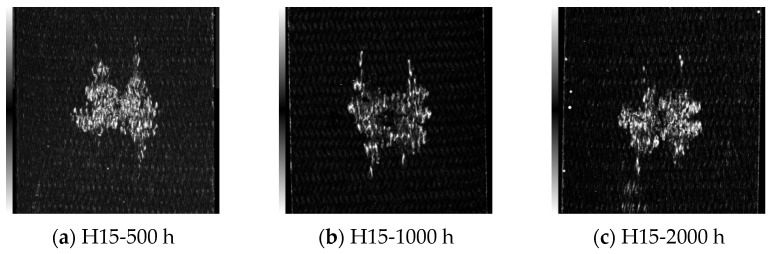

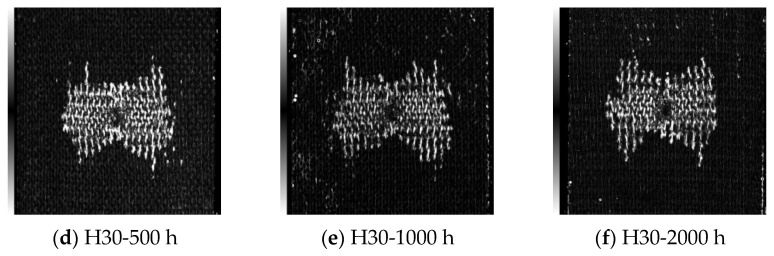
Internal damage of the specimen after LVI test under 70 °C-soak. The scanning area is uniformly 80 mm × 80 mm.

**Figure 12:**
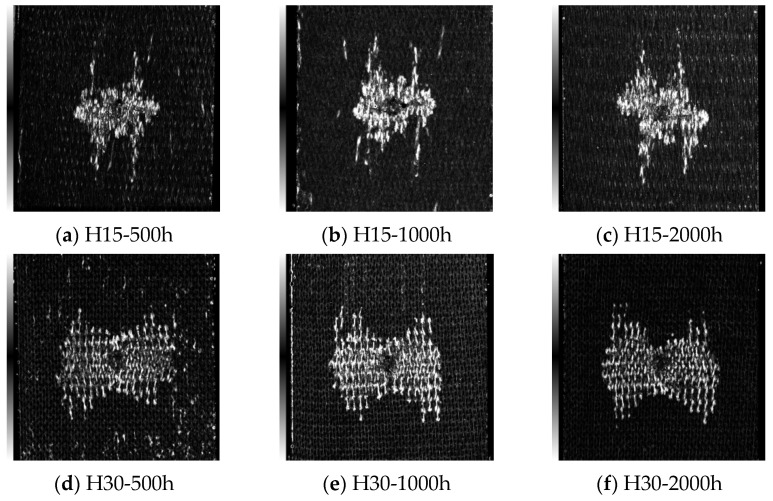
Internal damage of the specimen after the LVI test under 70 °C/85% RH. The scanning area is uniformly 80 mm × 80 mm.

Figure 9, Figure 10, Figure 11 and Figure 12 show the C-scan images of LVI tests in all cases. The white part in the figure shows the damaged area, which mainly results in fiber/matrix separation, fracture, and other damage. The overall picture shows that the damage area is H30 > H15, from large to small. It was found that the damage direction mainly extended along the knitting direction. With an increase in the braided angle, there is a butterfly extension trend from the center to both sides. Due to the smaller braided angle of H15 and the more dense arrangement of fibers, the trend of this butterfly damage is not obvious.

## 5. CAI Test and Experimental Results

### 5.1. Analysis of Impact Experiment Grouping Results

**Figure 13:**
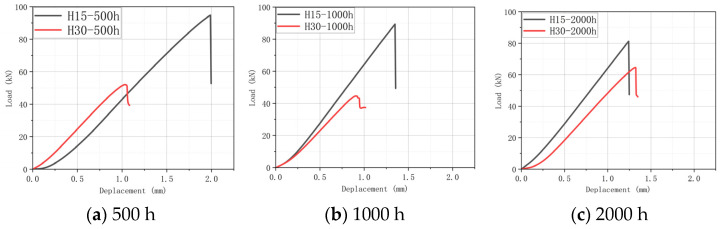
CAI test curve at 40 °C-soak.

**Figure 14:**
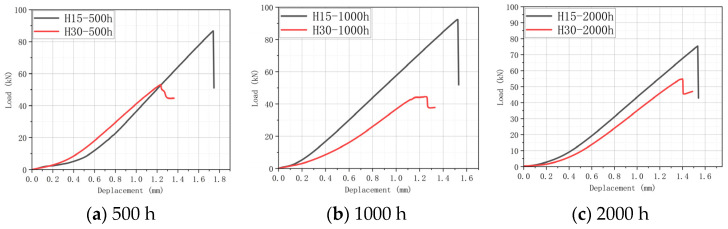
CAI test curve at 70 °C-soak.

**Figure 15:**
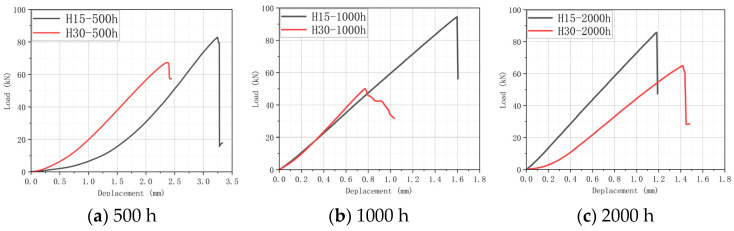
CAI test curve at 70 °C/85% RH.

The residual compression properties of composites are very sensitive to LVI damage. The residual compressive strength can reflect the damage tolerance of the composite to some extent. The CAI test was performed on all specimens to record the maximum load value (residual compressive strength load) during the compression process, as shown in Figure 13, Figure 14 and Figure 15. Table 6 shows the residual compressive strength loads under different aging conditions.

As can be seen in Table 6, the overall residual compressive strength is as follows: H15 > H30. This is due to the fact that H15 has a smaller braided angle and a denser carbon fiber arrangement, thus having a higher residual compressive strength. Based on the LVI test, it can be found that the smaller the braided angle, the lower the water absorption efficiency during the hygrothermal aging process, and the lower the impact peak load. Thus, the structural integrity is stronger, and residual compressive strength is higher.

**Figure 16:**
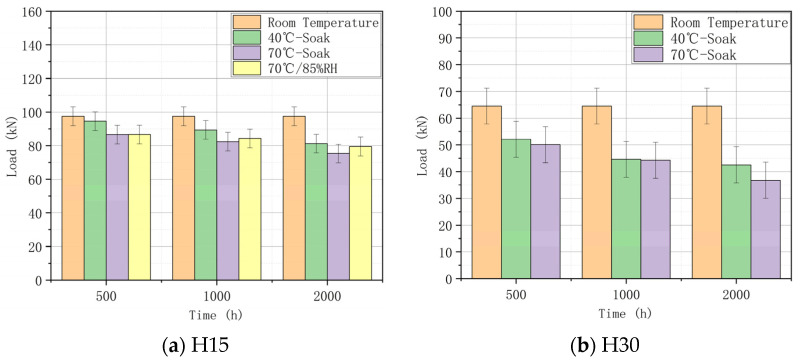
Residual compressive strength loads at different braided angles.

Statistical analyses of the residual compressive strength load of H15 and H30 are helpful for more visually displaying the change in their residual compressive strength, and the statistical results are shown in Figure 16. According to the LVI test, the following conclusions can be drawn: The hygrothermal aging process will lead to the deterioration of the integrity of the specimen, the impact resistance of the specimen is slightly improved, and the compression performance is reduced. In the LVI test, the impact of aging conditions on the impact resistance is 70 °C-85% RH > 70 °C-soak > 40 °C-soak. In the CAI test, the effect of aging conditions on the residual compressive strength is 40 °C-soak > 70 °C-soak > 70 °C-85% RH. The two groups of experiments further illustrate the correctness of their data.

### 5.2. Analysis of DIC Monitoring with Respect to the Damage Evolution Process

**Figure 17:**
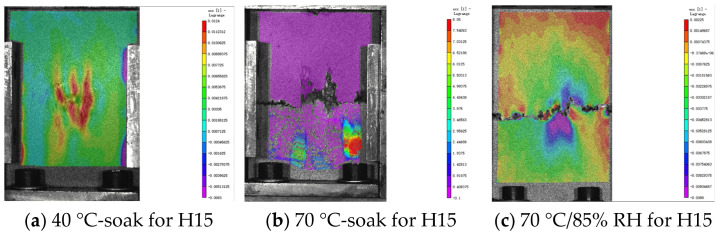

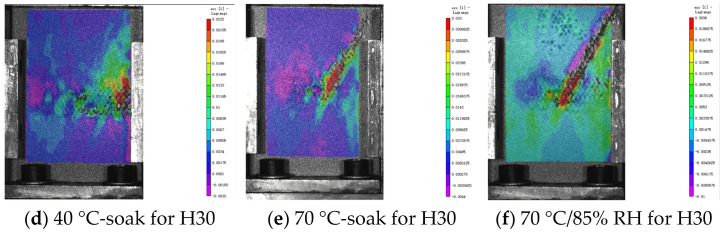
DIC results of the CAI experiment after 500 h of hygrothermal aging. The DIC monitoring area is 120 mm × 80 mm (specimen plane size). Please note that [1] in the figure does not represent a reference [1], it is a systematic display of the DIC processing software.

**Figure 18:**
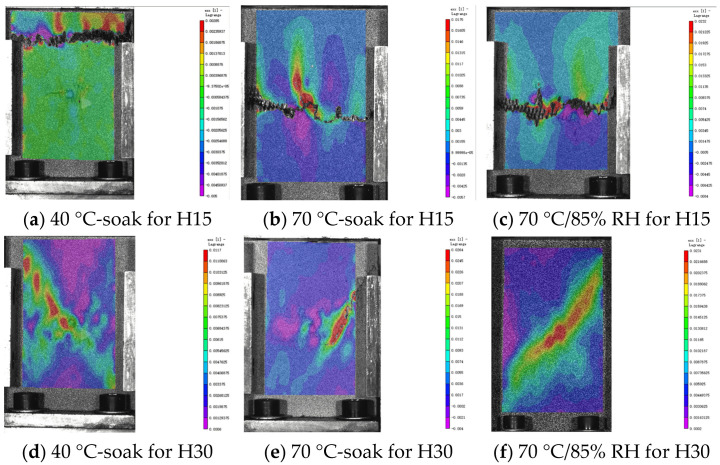
DIC results of the CAI experiment after 1000 h of hygrothermal aging. The DIC monitoring area is 120 mm × 80 mm (specimen plane size). Please note that [1] in the figure does not represent a reference [1], it is a systematic display of the DIC processing software.

**Figure 19:**
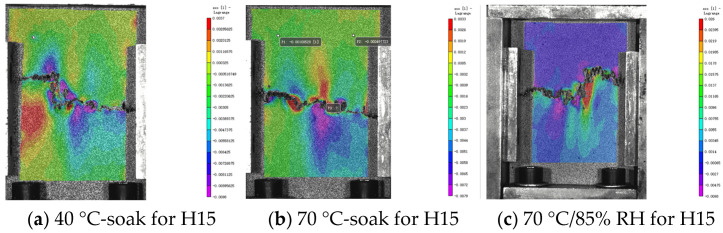

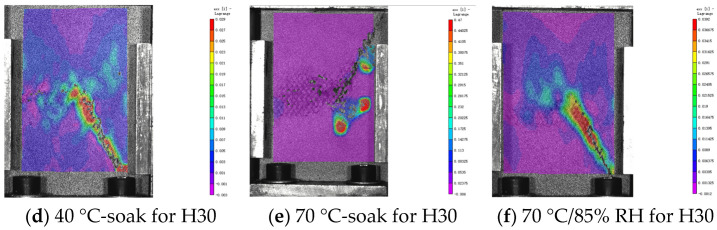
DIC results of CAI experiment after 2000 h of hygrothermal aging. The DIC monitoring area is 120 mm × 80 mm (specimen plane size). Please note that [1] in the figure does not represent a reference [1], it is a systematic display of the DIC processing software.

The damage development form of H15 and H30 during CAI was observed via DIC. The DIC results under different aging times are shown in Figure 17, Figure 18 and Figure 19. It should be noted that the DIC results presented during this time are all vertical displacement (V) results monitored via DIC. In the CAI test, H15 was accompanied by a crisp fracture sound, and the specimen was brittle and damaged. The fractured carbon fiber could be clearly seen at the fracture, and the shape of a fish scale appeared in the incomplete fracture. Plastic failure occurs in H30.

According to Figure 17, Figure 18 and Figure 19, the results show that the residual compressive load of the specimen decreases gradually with the aging time. Figure 17 shows that H15 and H30 fail at the impact point and extend through the impact point. There is stress concentration at the impact point. The failure fracture of H15 is extended in a horizontal manner and occurs laterally. The failure form is buckling failure, and brittle failure occurs. H30 occurs in the direction of 45°, and the overall fracture extends in an oblique upward manner. The failure mode is shear failure, and awakening failure occurs.

In Figure 18, the horizontal port of H15 does not appear in the middle but in the upper part. Since the fracture position does not penetrate the impact point, this may be due to the fact that the specimen is not completely vertically placed during the CAI.

The law shown in Figure 19 is the same as the above analysis.

## 6. Conclusions

In this paper, the effects of different hygrothermal aging conditions on the mechanical properties of 3D4d-BC with different braided angles were studied. TGA, SEM, C-scan, and other experimental methods were used to further characterize the hygroscopic behavior and damage morphology of the specimens. The mechanical properties were analyzed via the LVI test and CAI test. Through the above research, the research found the following:

(1) In the process of hygrothermal aging, a smaller braided angle leads to a lower equilibrium moisture absorption content. As a result, it has lower peak impact loads, greater structural integrity, and higher residual compressive strength;
(2) SEM images show that after the hygrothermal aging treatment, the oxidation and hydrolysis of the substrate surface will lead to the degradation of the substrate. Under complex stress conditions, microcracks will form on the surface of the matrix. This is the main reason for the degradation of the properties of composite materials due to the effect of hygrothermal aging;
(3) The intervention of oxygen molecules will degrade the properties of the fiber/matrix interface in the specimen and directly affect the residual compression performance of the specimen;
(4) The LVI test shows that temperature has a positive effect on the water absorption behavior of the epoxy resin matrix. Higher water absorption will improve the toughness of the specimen and improve the impact resistance of the specimen slightly. But at the same time, after the aging process of the specimen, the water absorption behavior of the matrix will further cause the separation of the fiber/matrix interface. This will lead to a reduction in the integrity of the specimen, and the residual compression property will also be reduced, affecting the strength and durability of the material
(5) The CAI test shows that the failure occurs from the impact point and extends to both sides through the impact point. The braided angle will cause different forms of compression failure. The brittle failure occurs in H15, the fracture occurs along the transverse, and the failure form is buckling failure. The plastic failure occurs in H30, the fracture occurs along a 45° direction, and the failure form is shear failure.

## Figures and Tables

**Table 1:** Main parameters of test materials.

Tape	Arguments
Carbon fiber	TORAY T700-SC-12000-50B
Matrix	TDE-86 epoxy resin matrix (H)
Fabric structure	Three-dimensional four-directional
Braided angle	15° and 30°
Yarn fineness	800 tex
Pitch length	41.0 ± 1.0 mm (15°) 21.5 ± 1.0 mm (30°)
Pitch width	11.0 ± 0.5 mm (15°) 12.5 ± 0.5 mm (30°)
Overall fiber volume content	(60 ± 4)%
Fiber filling factor	75%

**Table 2:** Name and model of the test equipment.

Equipment	Type	Main Parameter
Thermostatic water bath	HWY-501	Volume: 20 L; Temperature: room temperature −80.0 °C
Constant temperature and humidity testing machine	JHY-H-150L	Temperature interval: −60–150 °C Humidity interval: 20–98% RH

**Table 3:** Name and model of the impact test equipment.

Model Number	Main Parameter
Instron-9250 HV	Maximum impact energy: 826 J
	Maximum impact velocity: 20 m/s
	Hammer head positioning accuracy: 0.1 mm
	Mass of the impact platform: 7.29 kg (mass of single head ball punch: 0.145 kg)
Sonoscan-D9500	Probe frequency: 5–400 MHz
	Sound width: 0.25 ns–1 μm
	Maximum scanning area: 333 mm × 312 mm
	X/Y-axis accuracy: ±0.5 μm
	Z-axis accuracy: ±45 nm

**Table 4:** Material numbering (“R” means room temperature environment; “RH” means relative humidity).

Number	15° Braided Angle	Number	30° Braided Angle
#1	H15-R	#11	H30-R
#2	H15-500-40 °C	#12	H30-500-40 °C
#3	H15-500-70 °C	#13	H30-500-70 °C
#4	H15-500-70 °C/85% RH	#14	H30-500-70 °C/85% RH
#5	H15-1000-40 °C	#15	H30-1000-40 °C
#6	H15-1000-70 °C	#16	H30-1000-70 °C
#7	H15-1000-70 °C/85% RH	#17	H30-1000-70 °C/85% RH
#8	H15-2000-40 °C	#18	H30-2000-40 °C
#9	H15-2000-70 °C	#19	H30-2000-70 °C
#10	H15-2000-70 °C/85%RH	#20	H30-2000-70 °C/85% RH

**Table 5:** The ratio of the internal damage area of the specimens to the scanning area after impact under hygrothermal aging.

Number	Damage Area Proportion/%	Number	Damage Area Proportion/%
#1	9.55	#11	15.91
#2	9.86	#12	17.15
#3	10.00	#13	18.18
#4	11.26	#14	18.64
#5	11.36	#15	17.45
#6	11.14	#16	18.64
#7	12.27	#17	19.21
#8	12.52	#18	17.86
#9	13.45	#19	19.01
#10	14.59	#20	22.23

**Table 6:** Residual compressive strength loads during compression experiments under different hygrothermal aging conditions.

Hygrothermal Aging Conditions	Residual Compressive Strength Load/kN	
	H15	H30
Room temperature	97.49	64.54
40 °C-soak—500 h	94.58	52.09
40 °C-soak—1000 h	89.32	44.61
40 °C-soak—2000 h	81.20	42.54
70 °C-soak—500 h	86.60	50.08
70 °C-soak—1000 h	82.40	44.27
70 °C-soak—2000 h	75.32	36.80
70 °C/85%RH—500 h	86.65	51.36
70 °C/85%RH—1000 h	84.24	50.51
70 °C/85%RH—2000 h	79.51	39.85

## Data Availability

Data are contained within the article.
